# Expression profiles of miR3181 and miR199a in plasma and placenta of virally suppressed HIV-1 infected Cameroonian pregnant women at delivery

**DOI:** 10.1371/journal.pone.0268820

**Published:** 2022-05-20

**Authors:** Livo F. Esemu, Honore Awanakam, Dieudonne Nanfa, Michael Besong, Idriss Tsayem, Celine Nguefeu Nkenfou, Jude Bigoga, Rose Leke, Sobngwi Eugene, Lishomwa C. Ndhlovu, Gabriel Ekali Loni

**Affiliations:** 1 The Biotechnology Center, University of Yaounde I, Yaounde, Cameroon; 2 Centre for Medical Research, Institute Medical Research and Medicinal Plant Studies, Yaounde, Cameroon; 3 Department of Biomedical Sciences, Faculty of Health Sciences, University of Buea, Buea, Cameroon; 4 Department of Biochemistry, University of Yaounde I, Yaounde, Cameroon; 5 System Biology Laboratory, Chantal Biya International Reference Centre, Yaounde, Cameroon; 6 Department of Tropical Medicine, Medical Microbiology and Pharmacology, John A. Burns School of Medicine, University of Hawaii at Manoa, Honolulu, Hawaii, United States of America; 7 Division of Infectious Diseases, Weill Cornell Medicine, New York City, New York, United States of America; University of Nebraska Medical Center, UNITED STATES

## Abstract

Human immunodeficiency virus (HIV)-1 infection during pregnancy reduces the transplacental transfer of protective maternal antibodies needed to confer immunity during early postnatal life. However, the mediation of MicroRNA in this dysregulation is not well understood MicroRNAs 3181 and 199a have been shown to mediate neonatal Fc receptor (FcRn)-like transmembrane antibody transfer and endocytosis respectively but their expression levels in the placenta and plasma in women living with HIV have not been extensively investigated. The objective of this study was to determine how the expression levels of miR-3181 and miR-199a in the placenta and plasma are affected in women chronically infected with HIV who are on antiretroviral therapy (ART) and are virally suppressed at delivery. In this pilot case-control study, plasma and placenta biopsies were obtained from 36 (18 HIV+ and 18 HIV-) Cameroonian women at delivery. MicroRNAs 3181 and 199a expression levels were measured using RT-qPCR, data was analyzed using SPSS22.0 and R 3.60, and p values below 0.05 were considered statistically significant. All the HIV-infected women were on known ART regimens and were virally suppressed. There was no significant difference in the levels of miR-3181 (p>0.05) in the placenta and plasma amongst HIV-infected and HIV uninfected women. The expression levels of miR-199a were significantly greater in the plasma compared to the placenta of HIV+ (p = 0.00005) and HIV- (p = 0.027) women. Moreover, there was a significantly higher (p = 0.02) level of miR-199a in the plasma of women with HIV and their uninfected counterparts. Linear regression models adjusted for systolic pressure showed no significant difference (p>0.05) in the levels of miR-199a and miR-3181 in both the placenta and plasma due to HIV infection. Our findings suggest that even though ART uptake and viral suppression might help in maintaining miR3181 and miR199a levels in the placenta of women with HIV at comparative levels to those of their HIV negative counterparts, the significantly higher levels of miR-199a in the plasma of women with HIV compared to the placenta might highlight lurking systemic dangers and requires further investigation.

## Introduction

The Human Immuno-Deficiency Virus (HIV-1) /Acquired Immune Deficiency Syndrome AIDS, remains amongst the three big diseases afflicting mankind. According to the UNAIDS 2020 global HIV statistics 38.0 million people were living with HIV-1, of which 1.7 million people became newly infected in 2019, including 150,000 children who are below 15 [1]. Although sub-Saharan Africa harbors only 30% of the world’s population, it disproportionately bore 70% of the global total burden of HIV in 2020. Women also disproportionately bear the burden of the HIV epidemic [1]. Each year 1.3 million HIV-1 infected women are estimated to become pregnant 90% of these women live in Sub-Saharan Africa [1]. In Cameroon, the national HIV prevalence in 2018 was 5.0% in women and 3.4% amongst pregnant women [2]. The rising number of children born to HIV-positive women is a group of growing Public Health concerns because children exposed to HIV *in utero* are at an increased risk of mortality, morbidity, and slower early growth than their HIV unexposed counterparts [3–5]. In fact infants born to mothers with HIV in sub-Saharan Africa are vulnerable to more severe forms of common childhood infections, including malaria [3–5]. Maternal antibody transfer to the fetus is an important mechanism that protects newborns during their first year of life [3, 4, 6, 7]. Immunoglobulins (Ig) IgG1 is the major antibody isotype to cross the human placenta [3, 5, 6] and is mediated by the neonatal Fc receptor (FcRn) expressed on syncytiotrophoblast cells via an endocytosis dependent pathway [4–7]. Our previous study related maternal HIV-associated hypergammaglobulinemia (HGG) to reduced transplacental transfer of antibodies specific to 3 malaria antigens up to 6.5 folds less than their uninfected counterparts [8]. Although maternal HIV-associated HGG is incriminated for this reduction in antibody transfer, the model remains speculative. MicroRNAs regulate a plethora of fundamental biological processes such as cell differentiation, signal recognition, and pathogen responses [9] by fine-tuning the transcriptome. Recent findings show that miR3181 regulates the expression of the FcRn gene *(fcgrt)* by interacting with its 3’ untranslated region (UTR) in liver cells [10]. It is also known that miR-199a and miR-199b regulate endocytic transport an important step in the transfer of antibodies from the mother to the fetus [11]. HIV-1 infection reduces the transplacental transfer of maternal antibodies but the mechanism remains unclear [4, 8, 12]. Even when neonates escape HIV-infection, *in utero* exposure to HIV, ART and HIV immune activation may cause changes in the placental natural environment originally conducive for fetal growth, development and protection [4, 12]. The distributions of these microRNA in bodily compartments and how HIV affects this distribution is unknown. A better understanding of mechanisms underlying FcRn mediated transplacental antibody transfer, and the factors that affect these, is thus crucial for the optimizing knowledge in predicting the fetal outcomes at delivery. miR-3181 is a regulator of FCGRT mRNA expression while miR-199a is a noninvasive biomarker for the trafficking of substances via endocytosis. Although many studies have investigated the relationship between HIV-1 and the transplacental transfer of antibodies, little is known about the precise role of microRNA involved in this process in pregnant women infected with HIV-1. We sought to determine if levels of miR-199a and miR-3181 that play vital roles in the transplacental transfer of antibodies are affected in the plasma and placenta of virally suppressed expectant mothers with HIV-1.

## Methodology

### Ethical considerations

This study was nested under the umbrella PREVENT-IT study previously described by Loni and collaborators [13] under Clearance No 2017/02/873/CE/CNERSH/SP of the Cameroonian National Ethics Committee. This specific study was reviewed and approved by the Centre Regional Ethics Committee of Cameroon (Ethical clearance No.2018/1/CE0004/CRERSHC/) and IRB #STUDY00003832 of the University of Washington. All procedures were carried out according to the Helsinki declaration. Written informed consent was obtained from each participant before enrolment into the study. Confidentiality of participants was respected and databases only contained identifiers. The respondents were only identified by registration numbers instead of names.

### Study site and population

This pilot case-control study was carried out from July 2019 to December 2019 and nested in the PREVENT-IT study which aimed at investigating the effect of tenofovir and breastfeeding on the renal functions of HIV exposed but uninfected (HEU) children in Yaoundé Cameroon. The main study sites being the Antenatal Care (ANC) and maternities of the Efoulan, Cite-Verte district hospitals and Centre d’Animation Santaire et Sociale (CASS) Nkolndongo health facility all in Yaoundé. The study participants were Cameroonian women living in the city of Yaoundé, attending ANC and delivering at the maternities of these health facilities. The prevalence of HIV in the city of Yaoundé is 4.4% [2]. A total of 36 mother-neonate pairs were recruited in the study at delivery. Women with pre-existing health conditions [e.g., diabetes, preeclampsia and Hemolysis, and/or had spontaneous abortions] were excluded from the study. Information on each woman’s demographic and clinical history including HIV status, ART intake, use of the intermittent preventive treatment (IPT) with sulphadoxine-pyrimethamine (SP), and insecticide-treated bednets (ITN) during pregnancy was available. The birth weight, length, and Activity, Pulse, Grimace, Appearance and Respiration (APGAR) score of newborns were also available. Gestational age was estimated based on the date of the last menstrual period or ultrasound scan data when available. Neonates born between 28 and 37 weeks were classified as premature. Singletons weighing less than 2,500 grams were considered Low Birth Weight (LBW) babies. Women were tested for HIV during pregnancy and vaccinated with tetanus vaccine according to national guidelines. All HIV infected women were on ART following national guidelines.

### Sample collection

Maternal venous blood was collected in EDTA tubes immediately after delivery and placenta biopsies were washed three times in 1X PBS and stored in about 4-5mL RNA Later in 50ml falcon tubes, the venous blood was processed and preserved at -20˚C until analyses together with the placenta biopsies.

### MicroRNA isolation

We extracted microRNA from plasma and placenta tissues using the IBI extraction kit (IBI Scientific, Iowa USA, Cat IB47371) and mirVANA extraction kit (Life Technologies, California, USA, Cat AM1561) following the manufacturer’s protocol. Briefly, the RNA column was transferred into a new 1.5ml microcentrifuge tube. 50 μL of heated release buffer was added, incubated, and spun to obtain microRNA. Five μL of eluted microRNA was quantified using a NanoDrop spectrometer (Thermo Scientific, Wilmington, DE, USA). The quantity and quality of MicroRNA were measured as a proxy of those of total RNA using the Nanodrop Lite Spectrophotometer (Thermo Scientific, USA). Samples with ratio 1.8 to 2.2 qualified for downstream procedures.

### cDNA synthesis

cDNA synthesis from microRNA was done in a series of steps as described by the manufacturer. Briefly, poly (A) tailing was done using reaction mix in S1 Table in S1 File and under the conditions described on S2 Table in S1 File. Adaptor ligation was done following mix shown on S3 Table in S1 File and under conditions shown in S4 Table in S1 File. miRNA amplification was done using the mix on S5 Table in S1 File and under conditions in S6 Table in S1 File.

### Real-Time (RT)-PCR

Reagents were brought to ice, vortexed gently, briefly centrifuged and 1:10 dilution of cDNA template were prepared. RT-PCR mix was prepared as shown in Table 1.

**Table 1:** Components of the Real Time (RT)-PCR reaction.

Component	1 Reaction (μL)	36 Reactions (μL)
TaqMan^R^ Fast Advanced Master Mix (2X)	10	360
TaqMan^R^ Advanced miRNA Assay (20X)	1	36
RNase-free water	4	144
**Total PCR Reaction Mix volume**	15	540

Next, 15 μL of the PCR Reaction Mix were transferred into each well of the plate. Five μL of the diluted cDNA template were added to each well of the plate making a total volume of 20 μL. The reaction plate was sealed with an adhesive cover, then vortexed and centrifuged thoroughly to mix and spin down the contents respectively.

Using the QuantStudio^TM^ 5 user guide, detailed instructions about programming thermal-cycling or plate running were applied. The fast-cycling modes for the experiment were used and the following thermal-cycling conditions were set. The appropriate reaction volumes for each plate were set and ran as follows:

**Table:** 

Step	Temperature	Time	Cycles
Enzyme Activation	95°C	20 seconds	1
Denature	95°C	1 second	40
Anneal/Extend	60°C	30 seconds	40

### Absolute quantification of MicroRNA

The absolute amount of the 2 miRNAs in the plasma and placenta of HIV-infected and uninfected women was quantified. The absolute amount of each miRNA was calculated with respect to standard curves based on serial dilution (10^9^ to 10^3^ copies) of spiked synthetic mimics for miR-3181 and miR-199a-5p, using the same TaqMan microRNA assay according to the manufacturer’s protocol. The Ct values for each sample reaction were converted to the concentrations based on these standard curves.

The standards and the samples were then assayed in the same run.

### HIV RNA levels

HIV diagnostic data was available from the medical records at various health facilities. HIV copy numbers were determined for 18 HIV-1 (+) mothers who had enough peripheral plasma for testing at the National Public Health Laboratory, Yaoundé, Cameroon using the Abbott Real Time PCR HIV-1 kit with the m2000rt machine. Lower detection limits of the assay were less than 40 copies/mL; upper detection limit was above 10,000,000 copies/mL.

### Statistical analysis

MicroRNA levels, demographic and clinical variables were summarized using descriptive statistics: means and standard deviations or median and interquartile range (IQR), for continuous variables such as age or parity; and frequencies and percentages for categorical variables, e.g., maternal anemia status (yes or no) and HIV-1 infection status (yes or no). Two-sample t-tests or Mann-Whitney U-tests for continuous variables and Fisher’s exact tests for categorical variables were used to compare women with and without HIV-1. The expression levels of the markers were log-transformed into natural logarithm scales. The effects of maternal HIV-1 status on levels of each microRNA in each body compartment were evaluated through linear regression models, controlling for the selected demographic and clinical variables. All p values less than 0.05 were considered significant. All statistical analysis was performed using SPSS 22.0 and R statistical software v3.60. Linear regression models were used to analyze the effect of HIV-1 on placental and plasma microRNAs adjusted for systolic blood pressure.

## Results

### Participant characteristics

Demographic and clinical characteristics of study participants at delivery are summarized in Table 2. Overall, 36 women were enrolled in the study (18 HIV-1 positive and 18 HIV negative). HIV-1 positive and negative women were similar with respect to maternal factors: IPT use, temperature, blood pressure, peripheral malaria status, parity, and pregnancy outcomes: length of gestation, the proportion of singleton deliveries and C-section, neonate sex, neonate weight, and prevalence of LBW babies (all p-values>0.05). However, HIV-1 positive women had lower systolic blood pressure than their healthy counterparts (p = 0.02) with an average blood pressure of 112.94 ± 14.1 vs 128.78 ± 22.8 respectively. All (100%) of the HIV-1 positive pregnant women were receiving ART, and all of the women were on Tenofovir Lamivudine and Efavirenz tritherapy. HIV viral load was available for 18 (100%) and all participants were virally suppressed.

**Table 2:** Demographic and clinical parameters of mothers.

Characteristic	HIV-1(-)	HIV-1(+)	p-value
Number of enrolled participants, n	18	18	-
Age in years, mean ± SDθ	29.6 ± 6.3	30.72 ± 4.9	0.58
Maternal weight, mean ± SDθ	75.39 ± 14.2	72.78 ± 10.6	0.54
Maternal BMI in kg^˄^2, mean ± SDθ	28.55 ± 5.2	27.09 ± 3.7	0.34
ART use by pregnant women, n (%)	0	18 (100)	-
Maternal viral load, median (25^th^, 75^th^)	0	0(0, 150)	-
Maternal IPT use, n (%)	13(72.2)	8(44.4)	0.18
Number of SP doses pregnant women took, median, (25^th^,75^th^)	2(2,4)	1(0,2)	0.89
Maternal bed net use, n (%)	14(77.8)	15(83.3)	0.47
Maternal heart rate in beats per minute, mean± SDθ			
Systolic	128.78 ± 22.8	112.94 ± 14.1	**0.02**
Diastolic	78.72 ± 15.1	76.61 ± 13.9	0.67
Parity including current child, median (25^th^,75^th^)	3(1,4)	2(1,3)	0.33
Primigravidae, n (%)	3(16.7)	1(5.6)	0.37
Multigravidae, n (%)	15(83.3)	17(94.4)	0.37
Length of gestation in weeks, mean ± SDθ	39.67 ± 1.4	39.50 ± 0.9	0.68
Preterm deliveries	0	0	-

The data were summarized based on the non-missing values. The total % is not 100 due to missing values or values rounded. P-values were based on θ two-sample T-tests, π Mann-Whitney’s tests, F Fisher’s exact test.

There was no child born with low birth weight. Neonatal parameters were similar between babies born to women with and without HIV as shown in Table 3.

**Table 3:** Demographic and clinical parameters of neonates.

Characteristics	HIV-1(-)	HIV-1(+)	P-value
Singleton deliveries, n (%)	18(100)	17(94.4)	0.54
Male neonates, n (%)	11(61.1)	8(44.4)	0.42
Placental weight in g, mean± SD^θ^	617 ± 169	611 ± 151	0.73
Neonate weight in g, mean ± SD^θ^	3187 ± 348	3316 ± 326	0.26
Low birth weight, n (%)	0	0	-
APGAR at 1min, mean ± SD^θ^	8.72 ± 1.1	8.6 ± 1.2	0.78
APGAR at 5min, mean ± SD^θ^	9.28 ± 0.8	9.39 ± 1.0	0.71
Head circumference in cm, mean ± SD^θ^	33.89 ± 1.4	34.22 ± 1.7	0.52
Mid arm circumference in cm, mean ± SD^θ^	11.28 ± 0.8	11.56 ± 0.9	0.37

The data were summarized based on the non-missing values. The total % is not 100 due to missing values or values rounded. P-values were based on θ two-sample T-tests, π Mann Whitney tests, F Fisher’s exact tests.

### miR-3181 levels are not affected in plasma and placenta of virally suppressed HIV positive women

**Fig 1:**
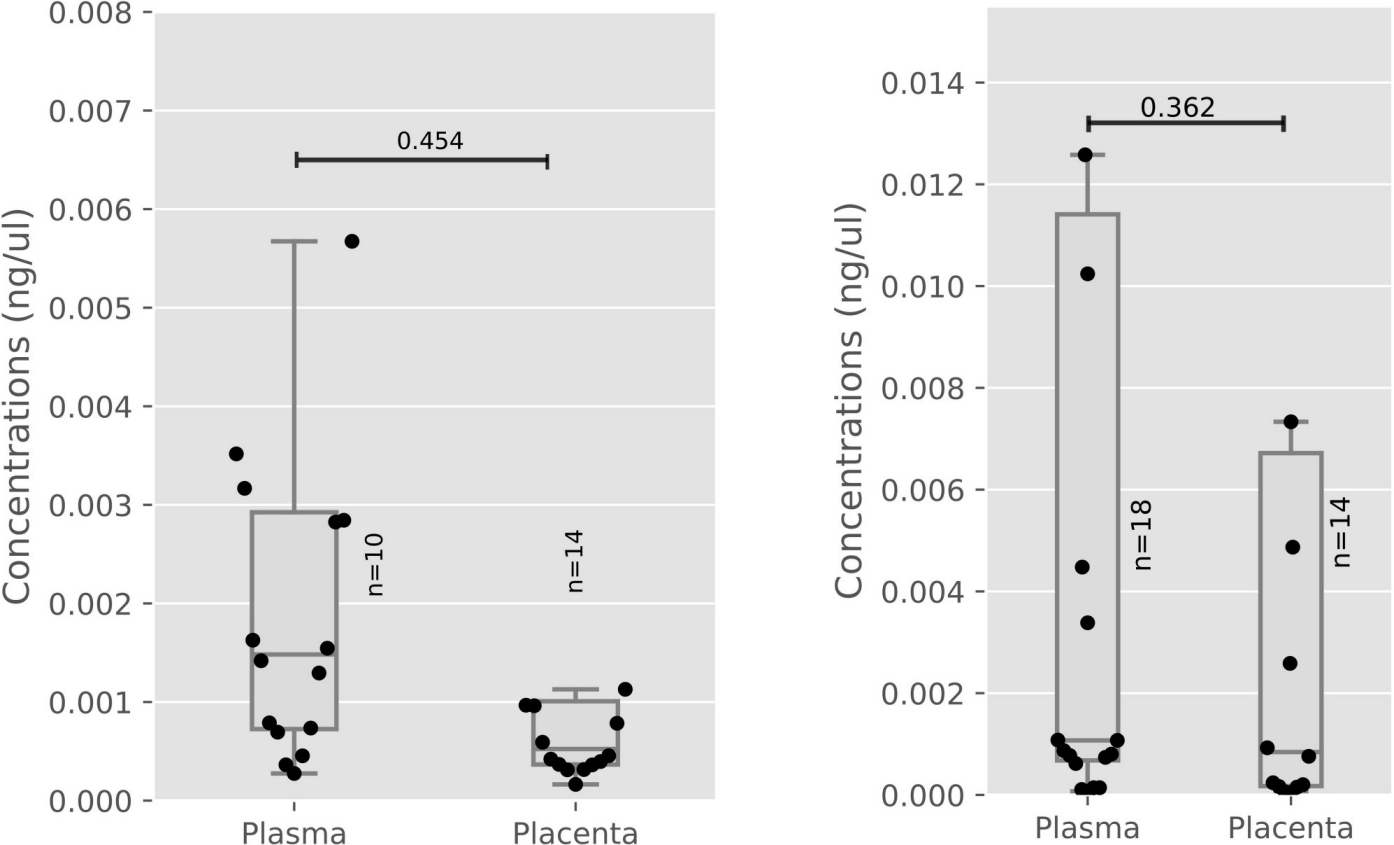
a: miR-3181 concentration (Plasma vs Placenta) in HIV Negative Women. b: miR-3181 concentration (Placenta vs Plasma) in HIV Positive Women.

The levels of miR-3181 were measured in the placenta and plasma. No significant difference (p = 0.45) in miR-3181 levels between the placenta and plasma in HIV-negative women was observed (Fig 1A). As well, Fig 1B shows there was no significant difference (p = 0.36) in the levels of miR-3181 between the placenta and plasma in HIV-positive women.

Fig 1A shows miR-3181 levels were measured in HIV Negative (plasma n = 10, placenta n = 14) women. Fig 1B shows miR-3181 levels were measured in HIV Positive (plasma n = 18, placenta n = 14) women. Median and interquartile ranges were plotted, differences between healthy and infected women were assessed using the Mann-Whitney test. HIV: Human immunodeficiency Virus, miR: microRNA.

**Fig 2:**
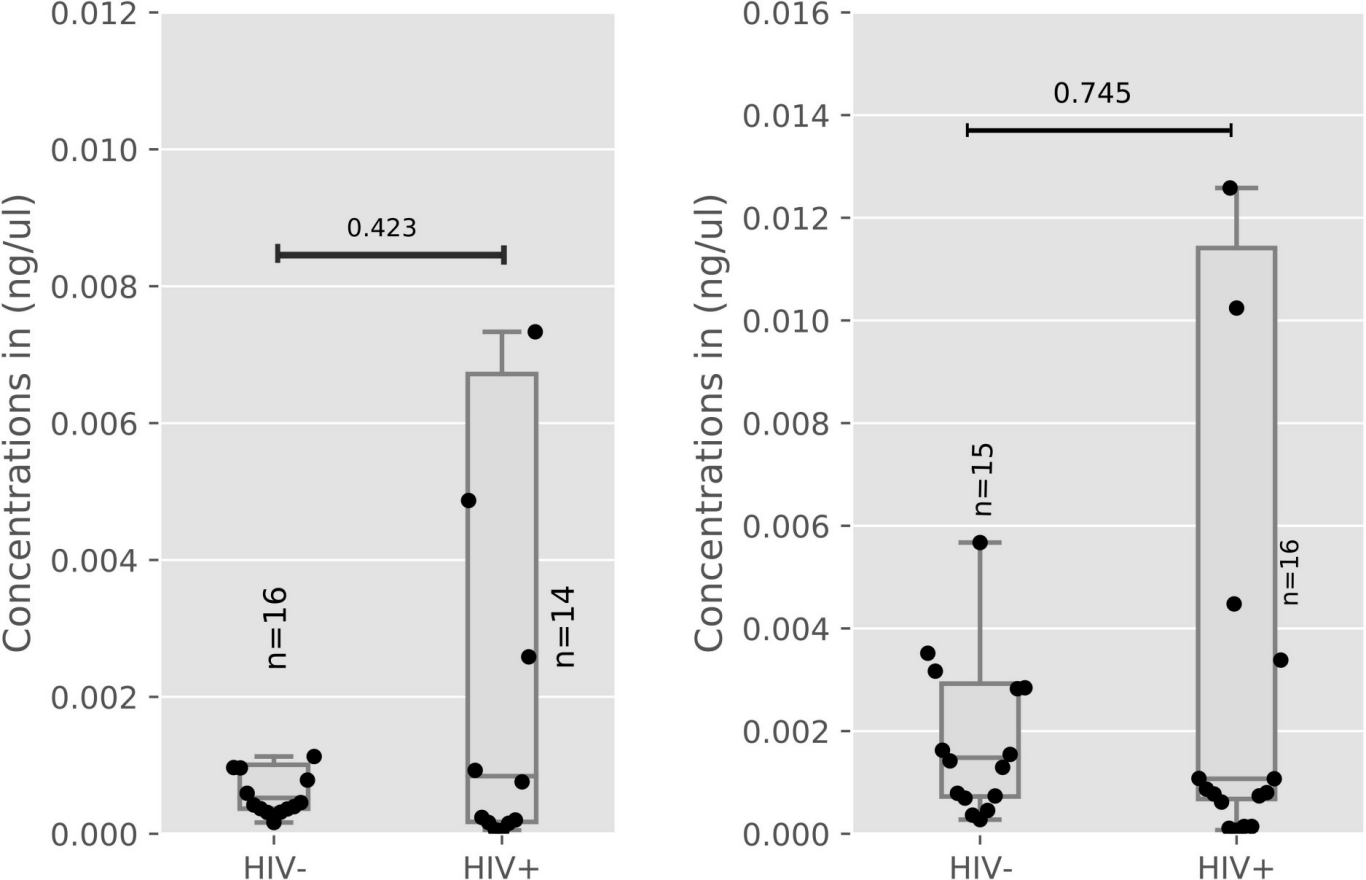
a: miR-3181 concentration in the Placenta of HIV Positive and Negative Women. b: miR-3181 concentration in the Plasma of HIV Positive and Negative Women.

On a similar note, levels of miR-3181 in the placenta of HIV-positive and negative women showed no significant difference (p = 0.42) as illustrated in (Fig 2A).

Fig 2B showed no significant difference (p = 0.74) in the levels miR-3181 in the plasma of HIV-positive women and their negative counterparts. Placenta to plasma ratios of miR3181 did not vary significantly between HIV + and HIV negative women as well as shown in S7 Table in S1 File.

Fig 2A exhibits miR-3181 levels were measured in the placenta (HIV- n = 16, HIV+ n = 14) between HIV-positive and negative women. Fig 2B shows miR-3181 levels were measured in plasma (HIV- n = 15, HIV+ n = 16) between HIV-positive and negative women. Median and interquartile ranges were plotted, differences between healthy and infected women were assessed using the Mann-Whitney test. HIV: Human immunodeficiency Virus, miR: microRNA.

### miR-199a levels are affected in plasma but not the placenta of virally suppressed HIV infected women

**Fig 3:**
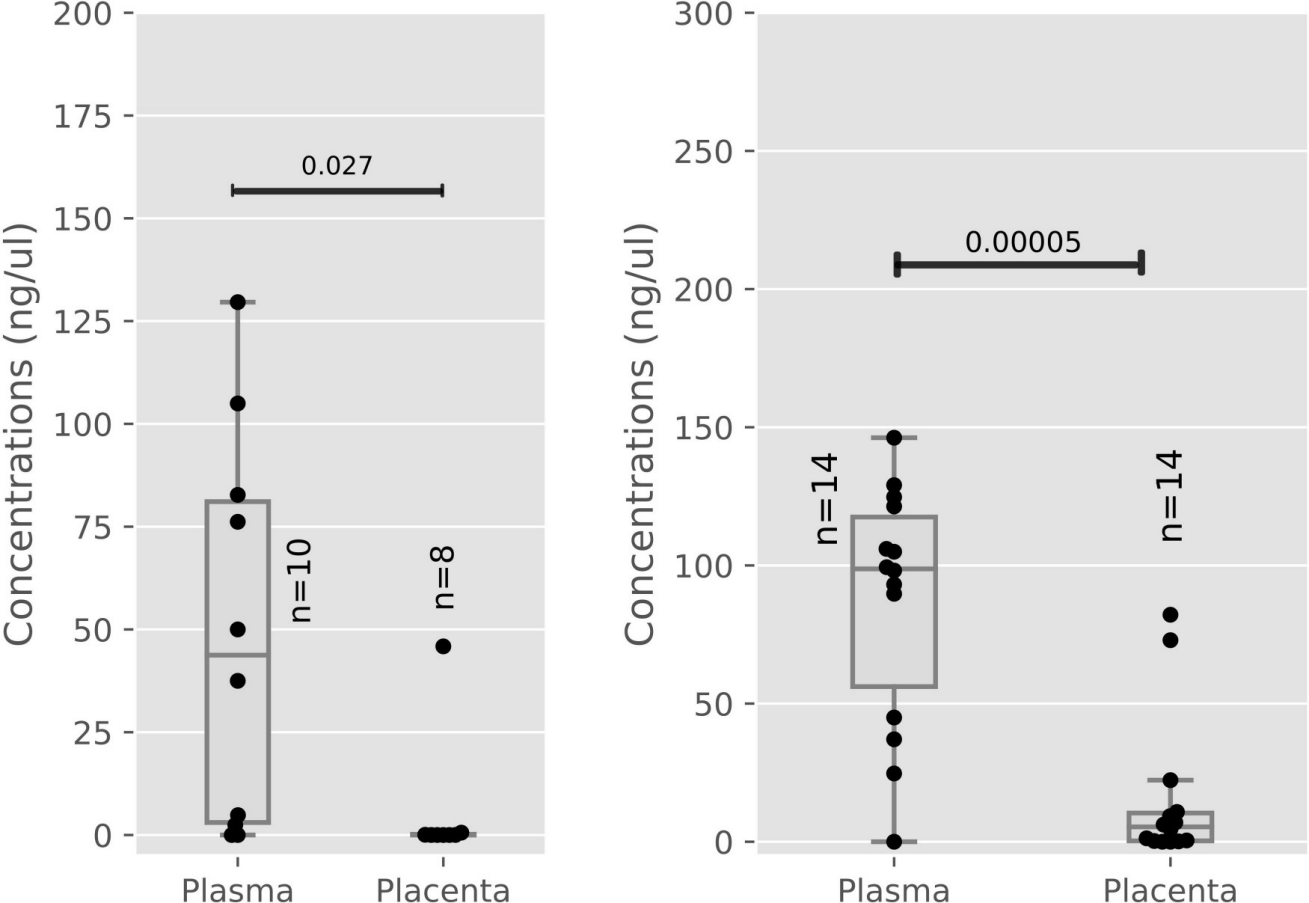
a: miR-199a concentrations (Placenta vs Plasma) in HIV Negative women. b: miR-199a concentrations (Placenta vs Plasma) in HIV Positive women.

There was a marked increase in the levels of miR-199a (p = 0.00005) in plasma of HIV-positive women compared to the placenta as shown in Fig 3B. A similar observation was made amongst HIV-negative women (p = 0.0275) as shown in Fig 3A.

Fig 3A indicates miR-199a levels were measured in HIV Negative (plasma n = 10, placenta n = 8) women. Fig 3B shows miR-199a levels were measured in HIV Positive (plasma n = 14, placenta n = 14) women. Median and interquartile ranges were plotted, differences between healthy and infected women were assessed using `the Mann-Whitney test. HIV: Human immunodeficiency Virus, miR: microRNA.

**Fig 4:**
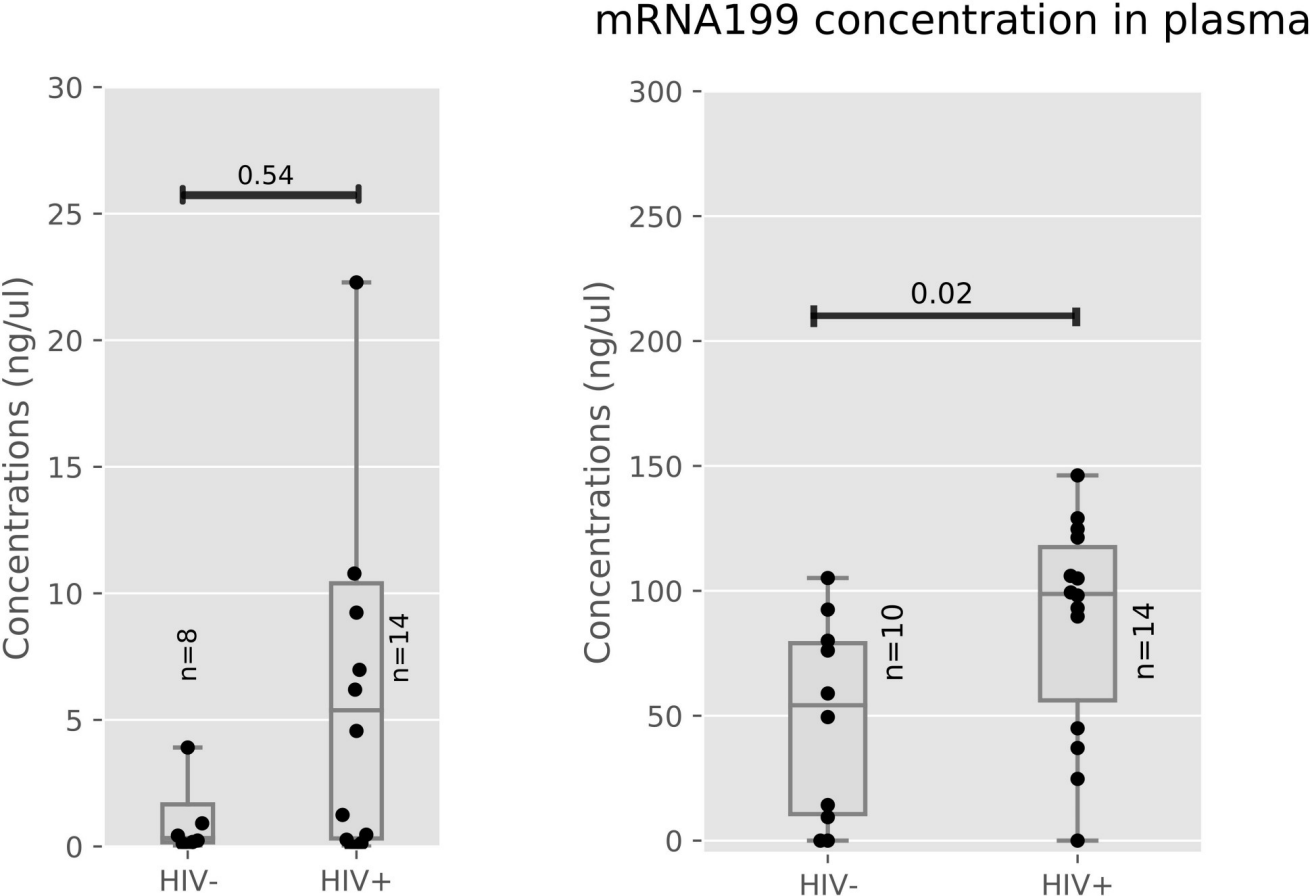
a: miR-199a concentrations in Placenta of HIV Positive and Negative women. b: miR-199a concentrations in Plasma of HIV Positive and Negative women.

Seemingly, levels of miR-199a in the placenta of HIV-positive and negative women were no significant differences (p = 0.54) as shown in Fig 4A. Rather a significant difference (p = 0.02) in the levels of miR-199a in the plasma was observed between the HIV positive and their negative counterparts (Fig 4B).

Placenta to plasma ratios of miRNA 199a was not significantly different between HIV-positive and Negative women (p<0.05) as seen in S7 Table in S1 File.

Fig 4A shows miR-199a levels were measured in the placenta (HIV- n = 8, HIV+ n = 14) between HIV-positive and negative women. Fig 4B portrays miR-199a levels were measured in plasma (HIV- n = 10, HIV+ n = 14) between HIV positive and negative women. Median and interquartile ranges were plotted, differences between healthy and infected women were assessed using the Mann-Whitney test. HIV: Human immunodeficiency Virus, miR: microRNA.

Linear regression models were used to analyze the effect of HIV-1 on placental miR-3181 and miR-199a levels. No significant differences (p = 0.51) for miR-3181 and miR-199a (p = 0.81) even when adjusted for systolic blood pressure as seen in Table 4.

**Table 4:** Placental microRNA level in HIV infection.

		HIV + vs HIV -		Systolic BP	
Biomarker	R^2^	Estimate (95% CI)	p-value	Estimate (95% CI)	p-value
miR3181	0.061	0.0754(-0.248, 0.399)	0.629	-0.0028 (-0.012, 0.006)	0.517
miR199a	0.007	0.0270 (-0.282,0.336)	0.855	-0.0008 (-0.008, 0.006)	0.816

The biomarkers levels in the placenta are transformed in a logarithmic scale and normalized. The model is adjusted for systolic blood pressure.

Linear regression models were used to analyze the effect of HIV-1 on Plasma miR-3181 and miR-199a levels, adjusted models for systolic blood pressure showed no significant differences either for miR-3181(p = 0.78) or miR-199a-5p (p = 0.75) levels due to HIV as shown in Table 5.

**Table 5:** Plasma microRNA level in HIV infection.

		HIV + vs HIV -		Systolic BP	
Biomarker	R^2^	Estimate (95% CI)	p-value	Estimate (95% CI)	p-value
miR3181	0.006	-0.0117 (-0.263,0.239,)	0.924	0.0009 (0.006,0.008)	0.780
miR199a	0.016	0.0671(-0.284,0.418)	0.688	0.0012 (-0.007,0.010)	0.753

*The biomarkers levels in plasma are transformed in logarithmic scale and normalized. The model is adjusted for systolic blood pressure.

## Discussion

Although a large number of studies have shown that several miRNAs participate in the development of cancer and other diseases [14], little is known about the role of miRNAs in the central cell biology process driving the transplacental transfer of antibodies. HIV-1 derived factors such as hypergammaglobulinemia have strongly been associated with the transplacental transfer of antibodies [12] howbeit biological processes involved with the antibody transfer have not been fully explored. This pilot case-control study used biomarkers important in the synthesis of FcRn (miR-3181) and the endocytosis (miR-199a), key processes in the transmembrane transfer of antibodies to assess changes in their expression levels in the placenta and plasma of pregnant women living with and without HIV. FcRn expression in the placenta regulates the concentration of IgG through their recycling and the process of transcytosis [15]. A study comparing the transfer of protective antibodies did not find any significant difference in the transfer of protective antibodies between women in monotherapy vs tri-therapy arms suggesting FcRn was not differentially expressed in both groups [16]. Here we used placental and plasma levels of miR-3181 as an inverse surrogate of FCGRT expression levels and microRNA 199a as an inverse surrogate marker for endocytosis efficiency.

We observed no significant difference (p = 0.36) in levels of miR-3181 between the placenta and plasma of HIV-positive women. It is plausible to speculate that suppressed viral load and antiretroviral therapy has no influence on transplacental antibody transfer converse to [7] who reported that women on long-term antiretroviral therapy had suboptimal transplacental antibody transfer.

No significant difference in miR-3181 levels (p = 0.45) was observed between the plasma and placenta of HIV-negative women. MicroRNAs might vary or not in different cellular environments in HIV-negative women. This is similar to reference [17] whose findings indicated that plasma and placenta microRNAs might play different roles in different cellular compartments., Their roles might nonetheless differ in different environments despite their levels being similar.

The marked difference (p = 0.00005) observed in the levels of miR-199a between the placenta and plasma in HIV-positive women might result from higher dysregulations in synthesis and trafficking of this microRNA across bodily compartments through exosomes [18].

Although discrepancies in the level of miR-199a in plasma and placenta in HIV-negative women were not as high as their HIV-infected pregnant counterparts, the difference was significant (p = 0.027). This could mean although women with HIV might be virally suppressed, they could potentially face dysfunctions in the endocytic process in other body compartments. Other findings suggest that in circulation, miR199a might play various immune regulatory roles in various body tissues [19]. However, it would be interesting to investigate their endocytic abilities in trafficking antibodies in these tissues.

No significant difference was observed (p = 0.54) in miR-199a levels in the placenta between HIV-positive and negative women. miR-199a is differentially expressed in different tissues playing varied physiological roles. Although the mechanisms underlying their roles in transplacental antibody transfer remain to be extensively studied, Gu et al. [20] as well as Morales-Prieto et al. [21] showed varied observations in their studies where miR-199a is deregulated primarily during tumorigenesis and hepatitis and equally plays an important role in the regulation of COX-2 expression in pregnant myometrium by blocking overexpression of TNF -alpha-induced myometrial cell contractility.

A significant difference (p = 0.028) in levels of miR-199a was observed in the plasma of HIV-positive and negative women. Lower levels in the plasma of HIV negative are likely due to lesser lymphocyte activation in the milieu of chronic immune stimulatory environment as variations in blood subpopulations can alter plasma miRNA levels. Although we did not do any correlation with CD4+ T cell numbers, Levine and collaborators [22] showed that lower levels of miRNA 199a may be associated with a decline in purified T and B lymphocytes from patients with primary Sjogren’s syndrome.

There were no significant differences in the levels of miR-3181 in the placenta (p = 0.42) and plasma (p = 0.74) between HIV-positive and negative women. This could imply that FcRn synthesis is not altered in virally suppressed HIV-positive women and the transfer of protective antibodies might be affected by other physiological processes and biomolecules. Nonetheless, findings by [23] showed how placenta inflammation led to an increase in threshold levels of unbound maternal antibodies causing saturation of placenta FcRn receptors available for efficient antibody transfer. These suggestions remain hypothetical.

A significant difference (p = 0.02) was observed in the systolic pressure between HIV-positive pregnant women and their healthy counterparts. Although this might translate residual heart conditions in HIV-1 infected women. These findings need more statistical power to be ascertained. These findings differ from those of [24] who found lower but not significantly different systolic pressure in HIV-positive patients in their study. Social and demographic parameters were homogeneous in neonates born to both HIV-positive and negative women. Linear regressions model showed that HIV did not independently affect miR-199a and miR-3181 levels and neither did it affect systolic pressure independently. Systolic pressure was also shown not to be independently affected by levels of miR-199a and miR-3181. We acknowledge a few shortcomings in this study like, the sample size was subjectively calculated and the study might be underpowered to make a resolute conclusion *sensus stricto*. However, the strongly discording levels of some markers (miR199a) observed between placenta and plasma of both HIV positive and negative women warrants further attention. Moreover, histological analyses were not performed to provide us with a picture of case-to-case links between miRNA levels and morphological abnormalities in these samples. Nonetheless, a study with a similar study population conducted in neighboring sites showed little morphological differences in the placenta of HIV-positive women and HIV-negative women [24]. Moreover, the sociodemographic, clinical, gynecological, and obstetrical differences between HIV-positive and negative women in the two studies showed a similar trend.

## Conclusion

Our findings suggest that even though ART uptake and viral suppression might maintain miR3181 and miR199a in the placenta of women with HIV at comparative levels to those of their HIV negative counterparts, the significantly higher levels of miR-199a in the plasma of women with HIV compared to the placenta might highlight lurking systemic dangers and requires further investigation. This implies that the reduction in the transplacental transfer of protective antibodies observed in women with HIV could be explained by other mechanisms.

## Supporting information

S1 File (DOCX)

S1 Database Mothers database. (XLSX)
