# Supplementary material for: Expression profiles of miR3181 and miR199a in plasma and placenta of virally suppressed HIV-1 infected Cameroonian pregnant women at delivery
Source: PLoS One. 2022 May 20;17(5):e0268820. doi: 10.1371/journal.pone.0268820 (PMC9122233; doi:10.1371/journal.pone.0268820)
Supplement: S1 File — (DOCX) [file pone.0268820.s001.docx]

**S1 Table: Components of Poly tailing mix**

| **Component** | **1 Reaction (**μ**L)** | **36 Reactions (**μ**L)** |
| --- | --- | --- |
| 10X Poly(A) buffer | 0.5 | 19 |
| ATP | 0.5 | 19 |
| Poly(A) enzyme | 0.3 | 6.4 |
| RNase-free water | 1.7 | 63.6 |
| **Total poly(A) reaction mix volume** | **3.0** | **108** |

The poly(A) reaction mix was briefly vortexed and spun, 2 μL of sample and 3 μL of poly(A) reaction mix was added to each well of the reaction plate to make a total volume of 5 μL. The reaction plates were then vortexed and centrifuged to spin down the contents and eliminate air bubbles, and then placed into the thermal then incubated using the following setting and standard cycling as shown below on S2 Table.

**S2 Table: Reaction condition for Poly tailing**

| **Step** | **Temperature** | **Time** |
| --- | --- | --- |
| Polyadenylation | 37°C | 45 minutes |
| Stop reaction | 65°C | 10 minutes |
| Hold | 4°C | Hold |

**S3 Table: Components of the adaptor ligation mix**

| **Component** | **1 Reaction (**μ**L)** | **36 Reactions(**μ**L)** |
| --- | --- | --- |
| 5X DNA Ligase Buffer | 3 | 108 |
| 50% PEG 8000 | 4.5 | 162 |
| 25X Ligation Adaptor | 0.6 | 21.6 |
| RNA Ligase | 1.5 | 54 |
| RNase-free water | 0.4 | 14.4 |
| **Total Ligation Reaction Mix volume** | **10** | **360** |

The ligation reactions were vortexed and briefly centrifuged. Next, 10 μL of the ligation reaction mix were added into each reaction plate containing 5 μL of the poly(A) tailing reaction product making a total volume of 15 μL per well. The reaction plates were sealed, vortexed, and centrifuged briefly to spin down the contents, later placed into a thermal cycler and incubated using the following settings on S4 Table below:

**S4 Table: Reaction conditions for ligation reaction**

| **Step** | **Temperature** | **Time** |
| --- | --- | --- |
| Ligation | 16°C | 60 minutes |
| Hold | 4°C | Hold |

**S5 Table: Components of the miR-Amp Reaction**

| **Component** | **1 Reaction(**μ**L)** | **36 Reactions(**μ**L)** |
| --- | --- | --- |
| 2X miR-Amp Master Mix | 25 | 1025 |
| 20X miR-Amp Primer Mix | 2.5 | 102.5 |
| RNase –free water | 17.5 | 717.5 |
| **Total miR-Amp Reaction** **Mix volume** | 45 | 1845 |

45 μL of it was transferred into each well of a new reaction plate. Five μL of the RT reaction product were added to each reaction well making a total volume of 50 μL. The reaction plates were sealed, vortexed, and centrifuged to mix and spin down the contents briefly. Placing the reaction plate in a thermal cycler at max ramp speed, the following settings were used on S6 Table.

**S6 Table: Reaction conditions for the miR-Amp Reaction**

| **Step** | **Temperature** | **Time** | **Cycles** |
| --- | --- | --- | --- |
| Enzyme activation | 95°C | 5 minutes | 1 |
| Denature | 95°C | 3 seconds | 14 |
| Anneal/Extend | 60°C | 30 seconds |  |
| Stop reaction | 99°C | 10 minutes | 1 |
| Hold | 4°C | Hold | 1 |

**S7 Table: Comparison of Placenta to Plasma ratios of miR3181 and miR199a in HIV positive and negative women.**

|  | HIV negative | | HIV positive | |  | |
| --- | --- | --- | --- | --- | --- | --- |
|  | **Ratio P/P** | **95 % CI** | **Ratio P/P** | **95 % CI** | **Test statistic** | **p-value** |
| **miRNA3181** | 3.856338 | -3.951342 11.664019 | 0.491570 | -0.105443 1.088583 | -0.8555 | 0.3995 |
| **miRNA199a** | 0.119148 | -0.158077 0.396374 | 0.117053 | -0.034561 0.268668 | -0.0164 | 0.9871 |

This table summarizes comparisons of ratio between placenta to plasma concentrations in HIV negative and HIV positive women. The test use for comparison is the Z-test for mean’s proportions in independent populations. There is no significant difference between groups, suggesting no statistically impactful difference between the P/P ratio in HIV negative versus HIV positive women both for mRNA3181 and mRNA199.

P/P = ratio Placenta / Plasma concentration
